# Characterization of mTOR Activity and Metabolic Profile in Pediatric Rhabdomyosarcoma

**DOI:** 10.3390/cancers12071947

**Published:** 2020-07-17

**Authors:** Luca Felkai, Ildikó Krencz, Dorottya Judit Kiss, Noémi Nagy, Gábor Petővári, Titanilla Dankó, Tamás Micsík, András Khoor, Tamás Tornóczky, Zoltán Sápi, Anna Sebestyén, Monika Csóka

**Affiliations:** 1 2nd Department of Pediatrics, Semmelweis University, 1094 Budapest, Hungary; felkailuca@gmail.com (L.F.); cartedorca@gmail.com (D.J.K.); 2 1st Department of Pathology and Experimental Cancer Research, Semmelweis University, 1085 Budapest, Hungary; krencz.ildiko@gmail.com (I.K.); n.noncsi@freemail.hu (N.N.); gaborpetovari@gmail.com (G.P.); tita.danko@gmail.com (T.D.); micsikt@gmail.com (T.M.); sapi.zoltan.dr@gmail.com (Z.S.); 3 Department of Laboratory Medicine and Pathology, Mayo Clinic, Jacksonville, FL 32224, USA; khoor.andras@mayo.edu; 4 Department of Pathology, Medical School and Clinical Center, University of Pécs, 7624 Pécs, Hungary; tornoczki.tamas@pte.hu

**Keywords:** mTOR, metabolism, rhabdomyosarcoma, pediatric

## Abstract

mTOR activation has been observed in rhabdomyosarcoma (RMS); however, mTOR complex (mTORC) 1 inhibition has had limited success thus far. mTOR activation alters the metabolic pathways, which is linked to survival and metastasis. These pathways have not been thoroughly analyzed in RMSs. We performed immunohistochemistry on 65 samples to analyze the expression of mTOR complexes (pmTOR, pS6, Rictor), and several metabolic enzymes (phosphofructokinase, lactate dehydrogenase-A, β-F1-ATPase, glucose-6-phosphate dehydrogenase, glutaminase). *RICTOR* amplification, as a potential mechanism of Rictor overexpression, was analyzed by FISH and digital droplet PCR. In total, 64% of the studied primary samples showed mTOR activity with an mTORC2 dominance (82%). Chemotherapy did not cause any relevant change in mTOR activity. Elevated mTOR activity was associated with a worse prognosis in relapsed cases. *RICTOR* amplification was not confirmed in any of the cases. Our findings suggest the importance of the Warburg effect and the pentose-phosphate pathway beside a glutamine demand in RMS cells. The expression pattern of the studied mTOR markers can explain the inefficacy of mTORC1 inhibitor therapy. Therefore, we suggest performing a detailed investigation of the mTOR profile before administering mTORC1 inhibitor therapy. Furthermore, our findings highlight that targeting the metabolic plasticity could be an alternative therapeutic approach.

## 1. Introduction

Rhabdomyosarcoma (RMS), the most common pediatric soft tissue sarcoma, counts for 3–4% of all childhood malignancies [1]. According to histological characteristics and different background pathomechanism, RMS can be classified into four main subtypes: embryonal (with the botryoid and anaplastic variants), alveolar, pleomorphic, and spindle cell [2]. Alveolar RMS is associated with the translocation of the Paired box (PAX) and the Forkhead box protein O1 (FOXO1) genes. The PAX-FOXO fusion-negative RMSs have a better prognosis than the fusion-positive ones. Besides the histological subtype, the prognostic factors are age, subtype, tumor size, site of origin, extension, and surgical resectability [1].

Regulatory failures lead to the activation of the phosphatidylinositol-3-kinase (PI3K)–protein kinase B (Akt)–mammalian target of rapamycin (mTOR) pathway which is common in a variety of sarcomas [3,4,5]. By enhancing anabolic processes, the mTOR pathway plays an important role in the regulation of cell proliferation, growth, and survival to fulfill the bioenergetic demands of tumor cells in response to environmental signals and intracellular conditions [3,6,7,8,9]. mTOR kinase is the centerpiece of the pathway and exists in two different complexes: mTOR complex 1 (mTORC1) and mTOR complex 2 (mTORC2), which differ in their protein components, cellular functions, and inhibitor sensitivity. The rapamycin-sensitive mTORC1 contains regulatory-associated protein of mammalian target of rapamycin (Raptor) as a characteristic scaffold protein and its activation results in the phosphorylation of S6 kinase (S6K). S6K phosphorylates ribosomal S6 protein, which leads to the promotion of protein synthesis, cell growth, and proliferation. Rapamycin-insensitive companion of mammalian target of rapamycin (Rictor) is a scaffold protein of mTORC2 and is considered to be resistant to rapamycin treatment. It phosphorylates Akt (at Ser473), serum and glucocorticoid-regulated kinase, and protein kinase C, thereby contributing to the regulation of actin cytoskeleton reorganization, cell migration, and survival [10].

Malignant cells rewire their metabolism to fuel the boosted cell cycle and tumor growth. The mTOR pathway is a key regulatory element of this metabolic adaptation, which is also linked to survival, metastasis formation, and changes in tumor microenvironment. Activated mTOR complexes can influence the metabolic profile by promoting glycolysis (including the Warburg effect, when cancer cells produce lactate instead of using oxidative phosphorylation even in aerobic conditions), lipid synthesis, pentose phosphate pathway, glutaminolysis, and the alteration of tricarboxylic acid cycle [11,12,13,14]. The expression of different metabolic enzymes can represent the metabolic characteristics of the tumor cell; however, little is known about these in RMS.

Similarly, the mTOR profile of RMSs has not been entirely characterized yet. However, mTOR pathway activation and anti-proliferative effects of mTORC1 inhibitors have already been reported in RMS cells in vitro [5,15,16,17]. In the ARST0921 clinical trial, a favorable event-free-survival was observed in relapsed patients who have received mTORC1 inhibitor temsirolimus in combination with conventional chemotherapy as compared to bevacizumab. These patients had not been screened for mTORC1/C2 activity before the treatment [18]. Unfortunately, several investigations could not find any significant effects of mTORC1 inhibitors on the prognosis of patients with RMS [19,20].

Despite the generally good prognosis, the survival rate of high-risk, metastatic, or relapsed patients is relatively low [4]. Biomarker-based patient selection has significant importance for personalized therapy, but mTOR complexes and the metabolic pathways have not been thoroughly analyzed in RMS. Therefore, we aimed to characterize the mTOR activity profile and certain metabolic alterations of patients with RMS in order to evaluate the potential efficacy of mTORC1/2 dual inhibitors. We also had the aim to find new opportunities for targeted therapy that can negatively affect metabolic adaptation, thereby enhancing the efficacy of current treatment options.

## 2. Results

### 2.1. Patients and Samples

We collected tissue samples of patients diagnosed with RMS between 2007 and 2017 (altogether 65 tissue blocks from 48 patients). Thirty-nine were categorized as embryonal, five as alveolar, and four as other subtypes. Alveolar RMS was considered as fusion-positive upon PAX/FOXO1 gene rearrangement and all other subtypes were considered as fusion-negative. The PAX-FOXO fusion status was determined as a part of the diagnostic procedure through fluorescence in situ hybridization (FISH) examinations (Vysis FOXO1 Break Apart FISH Probe Kit, 03N60-020, Abbott Laboratories) evaluating the signals in at least 100 tumor cells. Cases with break-apart signals in more than 20% of the cells were considered positive. The primary samples were taken at the time of diagnosis, and if available, specimens after preoperative chemotherapy and samples of the relapsed tumors were also studied. In addition, the expression profiles were compared in paired samples (primary–pretreated; *N* = 6 primary–relapse, *N* = 7; primary–pretreated–relapse, *N* = 2) of the same patients. Relevant clinicopathological data of the 48 patients are presented in Table 1 and Table S1. To assess IHC stainings, we calculated the histo-score (H-score) by multiplying the staining intensity (0, 1+, 2+, 3+) by the percentage of positive cells. Cases with an H-score higher than 100 were considered positive. We performed the statistical data analysis according to histological subtype, risk stratification, and survival as well (Table S2, Table S3).

### 2.2. The Expression of mTOR Activity-Related Proteins in RMS

To characterize the mTOR activity, phospho-mTOR (pmTOR) antibody was used, which recognizes the active form of the mTOR kinase. Furthermore, to distinguish mTORC1 and mTORC2 activity we chose two further immunostainings. mTORC1 activity was assessed by a phosphorylated ribosomal S6 protein (pS6) antibody. The amount of this phosphoprotein is in correlation with mTORC1 activity rather than the amount of Raptor, the scaffold protein of mTORC1 complex. mTORC2 phosphorylates Akt at serine 473; therefore, p(Ser473)-Akt could be used as a marker for mTORC2 activity. p(Ser473)-Akt immunostaining is highly dependent on sample handling, fixation (immediate fixation is necessary), and tumor size (e.g., how formalin infiltrates the whole specimen in large solid tumors), thus, we could not use it as a reliable marker of mTORC2 activity [21,22]. Instead of p(Ser473)-Akt, we analyzed the expression of Rictor (a scaffold protein of mTORC2) to evaluate the amount of mTORC2. Assessment of mTORC2 activity was based on the co-expression of pmTOR and Rictor. Rictor immunopositivity without pmTOR and pS6 positivity was considered as the potential activation of the mTORC2 complex [23,24].

**Figure 1:**
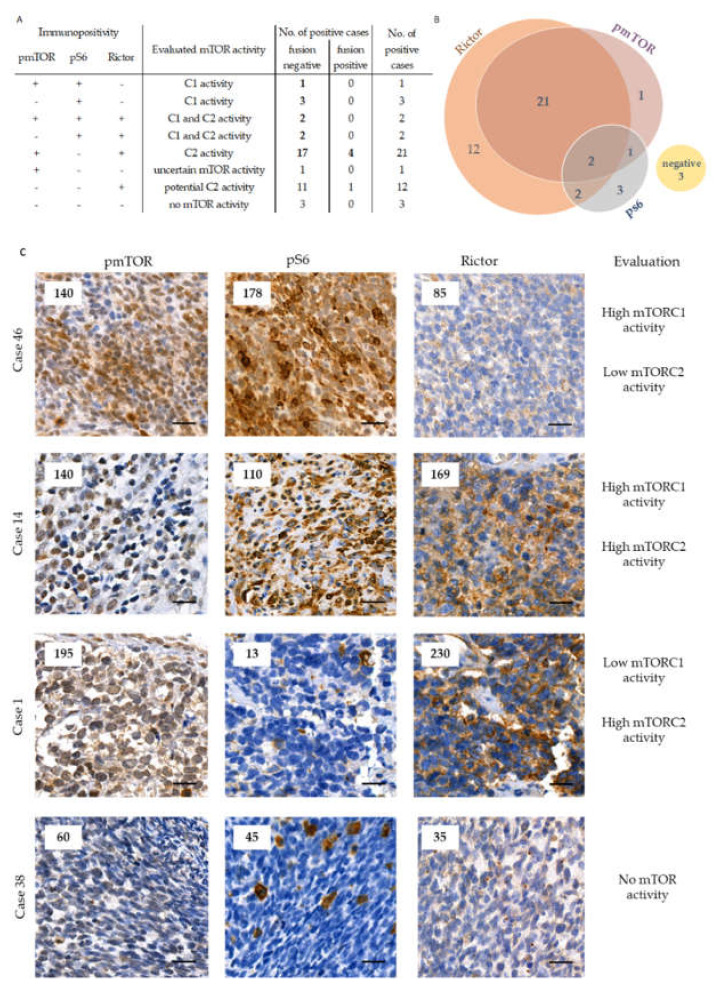
The distribution of the primary cases based on pmTOR, pS6, and Rictor immunostaining results and mTOR activity marker categorization. We present the results of the investigated mTOR markers with the interpretation of mTORC1 and/or C2 activity regarding PAX-FOXO fusion-negative and fusion-positive cases on the left (panel (**A**)). The cases with elevated mTOR activity were highlighted with bold numbers. The Venn-diagram (panel (**B**)) represents the number of positive cases with each immunohistochemical marker. On panel C representative pmTOR, pS6, and Rictor immunohistochemical stainings are shown for primary RMS cases (panel (**C**)). DAB (brown) and hematoxylin were used as chromogen and counterstaining, respectively. The numbers in the left upper corner of the pictures refer to the given H-scores. Pictures were taken with CaseViewer 2.3 software (3DHistech Ltd., Budapest, Hungary). The scale bars show 25 µm.

Most of the studied RMS cases showed detectable mTOR activity. The majority of the primary tissue samples were positive for pmTOR and/or pS6 (64%). mTOR activation was observed in 63% of the primary PAX-FOXO fusion-negative and in 80% of the primary fusion-positive cases (*p* = 0.020). pS6 stained faintly in most of the primary cases. The most interesting observation of the mTOR marker analysis was that Rictor expression was high in most of our primary samples (82%). Although the small number of cases weakens our results, all the studied fusion-positive (alveolar) cases had high Rictor expression (Figure 1, Table S2, Table S3).

**Figure 2:**
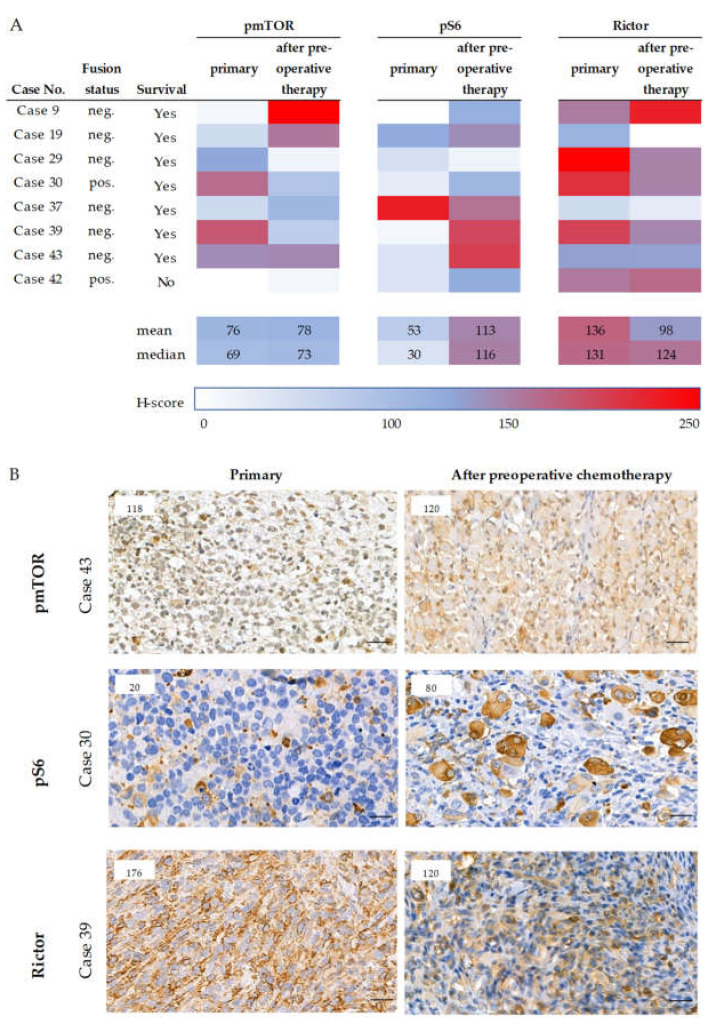
Expression of mTOR markers in paired samples (primary and pretreated). The H-score of mTOR related markers of the paired (primary and pretreated) cases are presented (panel (**A**)). The color scale labels the magnitude of the H-scores in each case. Survival data and the fusion status are also given. Under the table (**A**), representative pmTOR, pS6, and Rictor immunohistochemical stainings of primary and pretreated RMS cases are shown (panel (**B**)). DAB (brown) and hematoxylin were used as chromogen and counterstaining, respectively. The numbers in the left upper corner of the pictures refer to the given H-scores. Pictures were taken with CaseViewer 2.3 software (3DHistech Ltd., Budapest, Hungary). The scale bars show 25 µm.

In primary and pretreated tissue pairs pmTOR staining intensity did not change, but pS6 expression was higher in primary samples compared to pretreated cases (*p* = 0.003) (Figure 2). In contrast, Rictor expression decreased during treatment (*p* = 0.019), but considering all three markers (Rictor together with pmTOR and pS6 staining intensities), we cannot declare that chemotherapy significantly altered the mTOR activity in the analyzed primary and treated tissue sample pairs (Figure 2). We could find only one case (case 29) in which the expression of pmTOR, pS6, and Rictor also decreased in sarcoma cells after chemotherapy (Figure 2A). In accordance with this finding, this patient had an excellent clinical and histological response.

**Figure 3:**
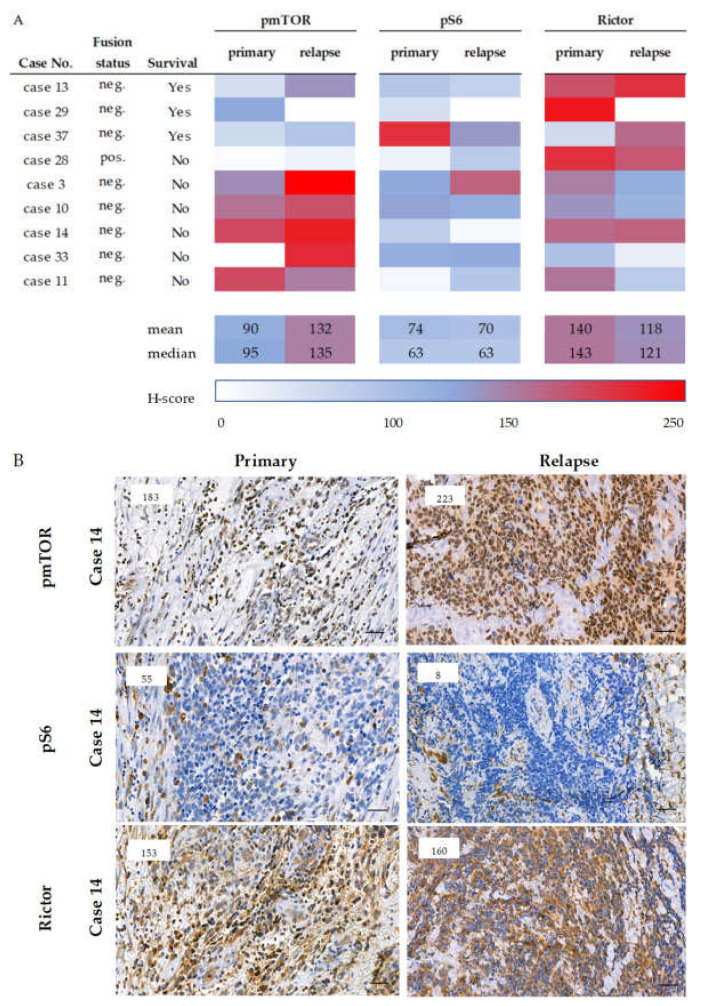
Expression of mTOR markers in primary and relapse paired samples. mTOR-related marker expressions of the paired (primary and recurrent) cases are presented (panel (**A**)). The color scale represents the H-score in each case. Survival data and the fusion status are also given. Under the table (**A**), representative immunostainings of mTOR activity-related markers of primary and relapsed RMS cases are shown (panel (**B**)). DAB (brown) and hematoxylin were used as chromogen and counterstaining, respectively. The numbers in the left upper corner of the pictures refer to the given H-scores. Pictures were taken with CaseViewer 2.3 software (3DHistech Ltd., Budapest, Hungary. The scale bars show 25 µm.

On the contrary, in the primary-relapsed sample pairs the recurrent sarcoma cells showed higher mTOR activity with an mTORC2 predominance compared to the primary biopsy material. The pS6 staining intensity remained low or decreased during relapse; however, the pmTOR scores were higher with a modest or remarkable increase in Rictor expression in the relapsed cases. The same changes were observed in both fusion-negative and -positive cases (Figure 3).

.

### 2.3. Analysis of Rictor Amplification in Rhabdomyosarcomas

According to the literature, elevated Rictor expression could be accompanied by *RICTOR* amplification [25]. In the selected 17 RMS samples with relatively higher Rictor protein expression (H-score ≥ 150), *RICTOR* amplification was studied as a potential mechanism for Rictor overexpression. These cases were compared to six samples with low Rictor expression (H-score < 100). According to the FISH analysis, the mean (range) copy number was 2.35 (1.33–4.70) in the studied RMS samples. There was no significant difference in the mean *RICTOR* copy numbers among the cases with low and high Rictor expression (2.39 vs. 2.33, respectively). Out of the 23 cases, one case was equivocal (with a *RICTOR* copy number of 4.70) and 22 cases were negative for *RICTOR* amplification. The presence or the absence of *RICTOR* amplification was further studied in the equivocal case using droplet digital PCR (ddPCR) copy number variant analysis. The *RICTOR*/AP3B1 ratio was 1.47; therefore, this case was also considered to be negative for *RICTOR* amplification (Table 2).

### 2.4. Expression of Metabolic Pathway-Related Proteins, Footprint of Warburg Phenotype, and Glutaminolysis in Rhabdomyosarcoma Cells

**Figure 4:**
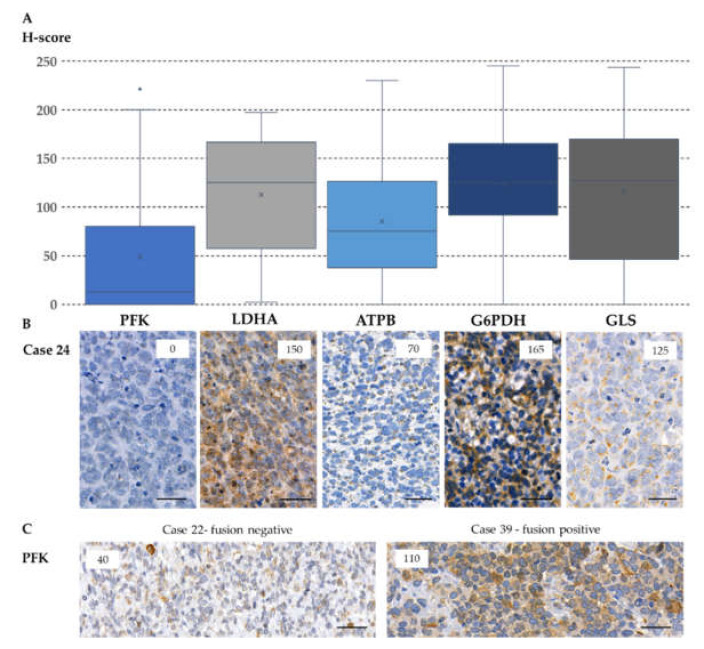
The metabolic marker expressions in primary rhabdomyosarcoma cases. The evaluation of metabolic markers in primary rhabdomyosarcomas showed that PFK and ATPB staining intensity remained mainly low; however, LDHA, G6PDH, and GLS stainings showed higher expression in most cases (**A**). Under the diagram, a representative case (case 24—a primary, fusion-negative case) is presented (**B**). The numbers in the right upper corner of the pictures refer to the given H-scores. All stainings are cytoplasmatic; however, ATPB and GLS show granular patterns due to their mitochondrial localization. DAB (brown) and hematoxylin (blue) were used as chromogen and counterstaining, respectively. Pictures were taken with CaseViewer 2.3 software (3DHistech Ltd., Budapest, Hungary), the scale bars show 25 µm. Metabolic enzyme expression profiles of fusion negative and positive cases differed only in PFK expression, which expressed at significantly higher level in fusion positive cases (*p* = 0.033), which were demonstrated in two representative cases (**C**).

**Figure 5:**
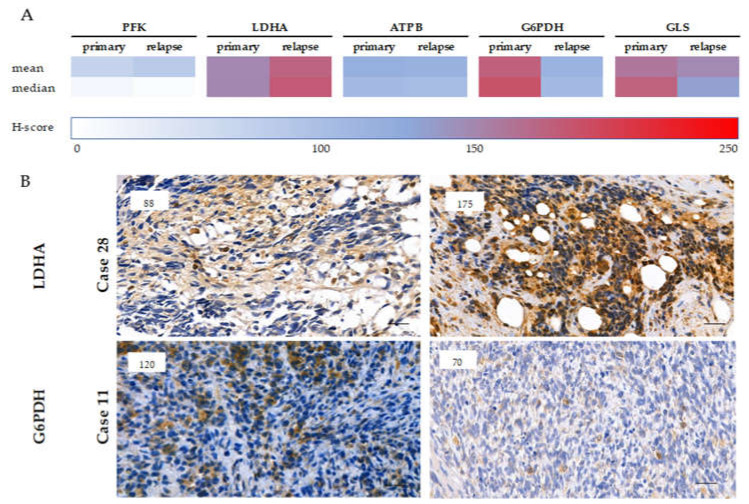
The expression of metabolic markers in paired (primary-relapse) cases. The color scales represent the medians and means of different metabolic marker H-scores in primary and relapse cases (**A**). LDHA and G6PDH immunohistochemical staining scores of primary and pretreated RMS cases showed remarkable alterations (**B**). The higher expressions of LDHA and the significantly decreased expressions of G6PDH in relapsed cases suggest the increased importance of Warburg-effect instead of pentose-phosphate pathway in relapsed RMSs. DAB (brown) and hematoxylin were used as chromogen and counterstaining, respectively. The numbers in the left upper corner of the pictures refer to the given H-scores. Pictures were taken with CaseViewer 2.3 software (3DHistech Ltd., Budapest, Hungary), the scale bars show 25 µm.

Cytoplasmic immunoreactivity of phosphofructokinase (PFK), lactate dehydrogenase-A (LDHA) (markers of glycolysis), and β-F1-ATPase (ATPB) (a marker of oxidative phosphorylation) was evaluated. We detected low expression of PFK in most cases (low staining intensity in 82% of the cases). Only the primary, PAX-FOXO fusion-positive cases showed increased PFK positivity in 60% of the cases. The difference in PFK expression between fusion-positive and -negative cases was significant (Figure 4 (*p* = 0.033)). In contrast, LDHA expression—the marker of Warburg phenotype, pyruvate-lactate transition—was high in most of the primary samples and even higher in recurrent cases (a remarkable, but not significant increase was detected in the median H-score, which needs further investigation—Figure 5). In RMS cells, we observed less pronounced oxidative phosphorylation in correspondence with low ATPB expression. These data suggest the importance of the presence of Warburg effect in RMS cells (Table S2, Table S3, Figure 4).

Evaluating glucose-6-phosphate dehydrogenase (G6PDH) as a marker of the pentose phosphate pathway, it was detected that 90% of the cases (out of primary samples) show high expression. In contrast, G6PDH expression was high in only 45% of the recurrent cases (*p* = 0.004). Therefore, our data suggest that the pentose phosphate pathway may have a crucial role in the development of primary tumors; however, it is less important in progression and relapse (Table S2, Table S3, Figure 4, Figure 5).

The expression of glutaminase (GLS) (a marker of glutaminolysis) was high in 71% of the primary and 55% of the recurrent cases, suggesting that glutaminolysis is rather implicated in the early steps of tumor development, using glutamine as an alternative bioenergetic substrate in RMS cells (Table S2, Table S3, Figure 4).

### 2.5. Correlation between mTOR and Metabolic Pathway-Related Proteins

**Figure 6:**
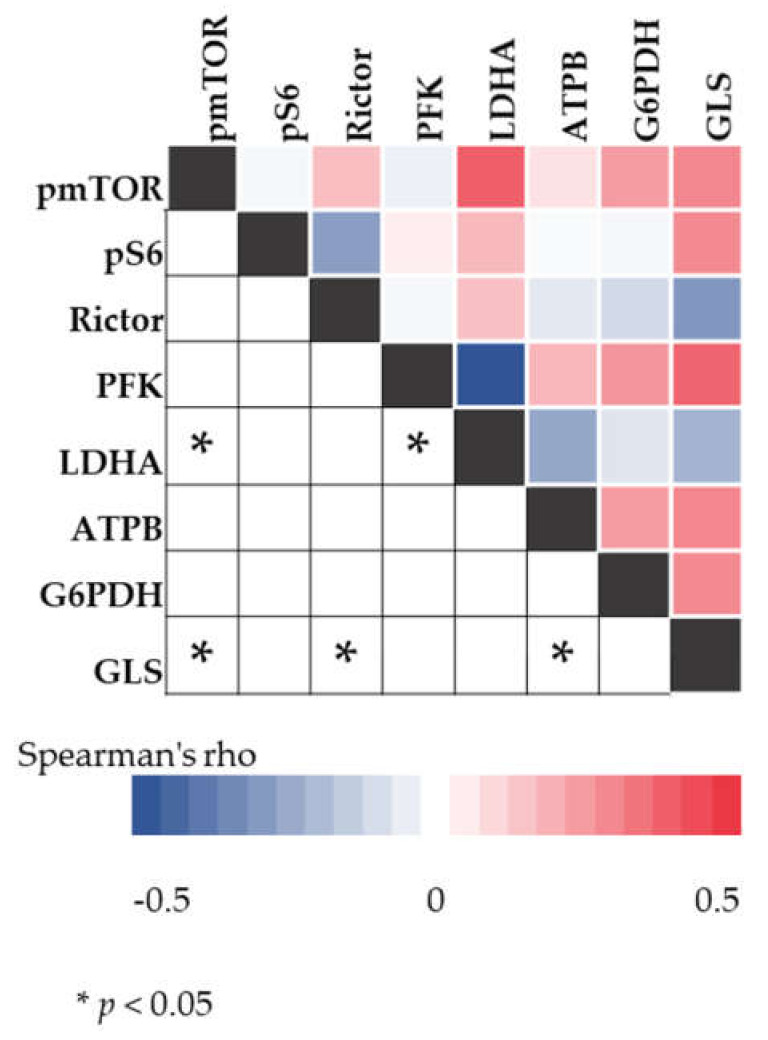
The results of the Spearman correlation analysis. The color intensity of heatmap changes according to the belonging Spearman’s rho value. The asterisks mark the significant (*p* < 0.05) correlations.

Data analyses with Spearman correlation test indicate associations between mTOR activity, LDHA and GLS expression in the studied cases. Positive correlations were found between pmTOR and LDHA expression (R = 0.403, *p* < 0.05) as well as between pmTOR and GLS expression (*R* = 0.302, *p* < 0.05) (Figure 6).

### 2.6. Survival and the Progression of a Studied Rhabdomyosarcoma Case

**Figure 7:**
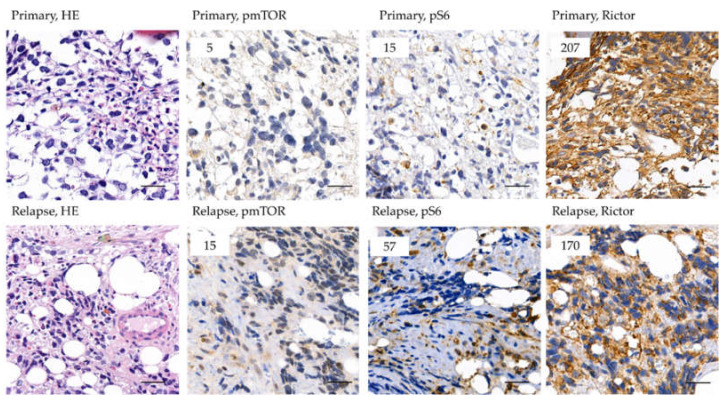
Expression of mTOR related markers of the patient treated with mTORC1 inhibitor. Representative areas of the immunohistochemical stainings (pmTOR, pS6, Rictor) are presented in the figure. The specimens belong to the patient (case 28), whose mTOR inhibitor therapy showed to be inefficient. The patient had multiplex bone metastasis and all his tissue samples were bone marrow biopsies. DAB (brown) and hematoxylin were used as chromogen and counterstaining, respectively. The numbers in the left upper corner of the pictures refer to the given H-scores. Pictures were taken with CaseViewer 2.3 software (3DHistech Ltd., Budapest, Hungary). The scale bars show 25 µm.

We present the case of a 10-year-old patient diagnosed with relapsed alveolar RMS. The second-line treatment (CWS 2012 guideline, relapsed protocol [26]) showed to be effective, but 2 months after the end of treatment, relapse was confirmed. Based on ALK positivity, we started ALK inhibitor treatment. Partial response was observed besides good clinical improvement, but further progression was confirmed 3 months later. According to some promising investigations documented in the literature, after moderate pS6 immunopositivity was confirmed, an mTORC1 inhibitor—temsirolimus—was added to his treatment. After a lack of therapeutic response, we lost the child within 3 months. During our recent investigations, the samples of this patient were further studied for detailed mTOR characterization. The primary and relapsed samples both showed low expression of pS6 (H-score 15 and 57); however, Rictor expression was high (H-score 207 and 170) in both cases (Figure 7, Case 28 in Figure 3). These data suggest mTORC2 dominance, which can be an explanation for the inefficacy of mTORC1 inhibitor therapy. The patient is presented as case 28 in our analysis (Figure 3).

## 3. Discussion

RMS is an aggressive soft tissue sarcoma in childhood. Patients with localized disease and good response to chemotherapy, especially with appropriate local control, have a good prognosis. However, the survival of relapsed patients or cases with continuous tumor progression remains quite poor. In relapsed cases, the treatment options are also limited by previous therapies and side-effects. To improve the outcome and diminish late side-effects, more effort is needed to define the molecular mechanisms driving RMS tumorigenesis and progression [4]. Therefore, we aimed to characterize the mTOR and metabolic profile of pediatric RMS cases as a potential target for personalized therapy.

Some preclinical data and certain clinical investigations with combined kinase inhibitors suggest the importance of mTOR activity in RMS cell proliferation and survival [27,28]. However, limited data are available about the in situ protein expression of mTORC1 and mTORC2 activity markers, including pS6 and pAKT. Some studies have described high pAKT expression in RMS cells [29,30]. Moreover, Petricoin et al. have found an association between mTOR activation and unfavorable clinical outcome [31]. Consistent with these findings, we observed high pmTOR expression in 58% of the studied tumors, suggesting that mTOR kinase activity is elevated in a significant proportion of RMS cases. In addition, a remarkable difference was found between PAX-FOXO fusion-positive and -negative cases (55% of the fusion-negative and 80% of the fusion-positive cases were positive for pmTOR). All primary fusion-positive cases showed strong Rictor positivity in this study. Moreover, we detected Rictor overexpression in more than 80% of the studied samples, whereas pS6 expression, a marker for mTORC1 activity, was low in most of the cases. Our results imply the hyperactivity of mTORC2, besides the less pronounced mTORC1 activity in the majority of the cases.

Characteristics and behavior of malignancies can change during chemotherapeutic treatment and progression. Therefore, we aimed to compare mTOR activity and certain metabolic enzyme expression in our paired samples. Two different groups of paired tumor samples were available for further evaluation: (1) diagnostic tumor material before treatment paired with samples after preoperative chemotherapy and (2) diagnostic tumor material paired with samples from recurrent patients. Our results suggest that chemotherapy did not cause a relevant decrease in mTOR activity. However, pS6 protein H-score increased after treatment; this elevation was detected due to the increase in the number of single-standing sarcoma cells with strong immunopositivity, which could be caused by the withdrawal of the used mitosis blocking agent (e.g., actinomycin-D). It has been described that actinomycin-D decreases the mTORC1-related ribosomal S6 kinase activity in tumor cells [32]. The withdrawal of such therapy before surgery can reload the proliferation and can overstimulate S6 phosphorylation. This potential role of the S6 kinase activity in growth of RMS needs to be further investigated. We also evaluated the recurrent samples in accordance with survival. The patients who could be cured from the relapsed disease showed low pmTOR levels both at the time of diagnosis and at the recurrence as well. However, patients with treatment failure showed higher pmTOR levels, and these values were raised with disease progression. The relatively higher initial pmTOR levels and their further increase during relapse were connected with worse prognosis in paired samples. In these finally incurable patients, the high pmTOR and the unchanged low pS6 levels were accompanied by high Rictor expression during disease progression.

Different mTOR pathway inhibitors are available for clinical use. The most widespread ones are the mTORC1 inhibitors of the rapalog family. Some previous studies have found that RMS cell lines could be sensitive to rapamycin. Hosoi et al. reported that rapamycin caused cell cycle arrest and apoptosis in human RMS cell lines Rh1 and Rh30 [33]. Despite the reported inhibitory effect of rapalogs in preclinical models, clinical trials have observed variable efficacy [16,17]. Some studies have not been able to confirm the expected response using rapalog inhibitor therapy [19,34]. Meanwhile, the results of the latest clinical trial suggest the benefit of additional temsirolimus therapy in comparison with bevacizumab [18]. Further improvement is expected via using dual inhibitors, since these can overcome rapalog resistance by targeting both the mTORC1 and the C2 complexes or the PI3K-mTOR pathway simultaneously [5,35,36,37]. Some investigations have reported the strong inhibitory effect of mTORC1/2 dual inhibitors in vitro as well. According to these, dual inhibitors—OSI-027 and PP242—caused catastrophic micropinocytosis in RMS cell lines RD and Rh30 [32]. It has also been described that MLN0128, a dual mTORC1/C2 inhibitor, induced apoptosis in vitro and in vivo in nine different RMS cell lines. Some controversial data are available about the mTOR inhibitor sensitivity in RMS cell lines. In contrast with the above-mentioned findings, Hosoi et al. described that rapamycin did not induce apoptosis according to their results [38]. As a further therapeutic option PI3K/mTOR (e.g., omipalisib, NVP-BEZ235) or selective PI3K inhibitors (e.g., alpelisib, buparlisib) can also be used. Several studies have reported promising results of in vitro investigations in RMS cell lines, suggesting the benefit of using these inhibitors to potentiate the effect of other antiproliferative agents [5,37,39,40,41]. Considering our immunohistochemistry results and our single case experience—in line with the previously reported findings—we suggest performing mTOR activity profiling in situ before starting mTOR inhibitor therapy. Analyzing patients’ biopsy materials could help to detect high mTORC2 activity or Rictor overexpression. In addition, it may predict resistance to rapalog and influence treatment selection.

Regarding to mTOR profile evaluation, we also studied the metabolic features of RMS cells. Tumor cells rewire their bioenergetic and biosynthetic pathways to support cell growth and survival, in which mTOR can play a key role [42,43,44]. We investigated certain glycolytic activity-related enzymes (PFK and LDHA) and other bioenergetic mechanisms, such as oxidative phosphorylation (ATPB), pentose phosphate pathway (G6PDH), and glutaminolysis (GLS). It has been published that RMS cells show a metabolic shift to altered glucose metabolism in in vitro models [45,46]. Based on these findings, the enhanced pentose phosphate pathway activity of RMS cells has also been suggested [45]. Elevated G6PDH expression in our primary samples underlines the role of pentose phosphate pathway and nucleotide biosynthesis in vivo in RMS tissue, as well. Additionally, we detected partially elevated LDHA levels, which increased during disease progression. This finding highlights the presence and the potential activation of Warburg phenotype-related enzymes in disease progression. The described expression profile of the investigated metabolic enzymes—low ATPB expression and high LDHA expression—emphasizes the lower importance of oxidative phosphorylation. In this context, preclinical investigations are controversial since both mitochondrial respiratory deficiency and intact function of the mitochondria have been reported [45,47]. Based on our results, we can conclude that RMS cells can have a metabolic shift to altered Warburg effect in situ in the tissue microenvironment. The characterization of lipid metabolism was not performed in our investigation. However, facilitated lipid degradation promotes cell survival and metastasis in a preclinical RMS model [48]. Additionally, the recently described in situ GLS overexpression in RMS samples suggests a high glutamine demand of in vivo tumor growth. The importance of our findings on glutamine dependency during progression is highlighted by previously published results on in vitro RMS cell line models [49]. In our study, the detected LDHA and GLS expressions showed correlation to pmTOR activity levels. These findings suggest that activated mTOR enhances the activity of LDHA and GLS, and through that, the anaerobic glycolysis and glutamine utilization. This observation has a special importance in metabolic adaptation of RMS cells and indicates that mTOR inhibitors can also affect bioenergetic processes, which may contribute to their therapeutic effects. This metabolic adaptation needs further investigation throughout different models, as these pathways differ in 2D, 3D cell cultures and in vivo experiments.

Metabolic plasticity can be mediated by several different mechanisms as Warburg effect, oxidative phosphorylation, glutaminolysis, and other substrate consumption (e.g., lipolysis). Investigating these concepts may lead to a better understanding of therapy resistance and new ways of targeting neoplastic cells through metabolic pathway alterations. mTOR complexes, as important regulatory elements of the cellular signaling network and metabolic regulatory proteins can orchestrate these mechanisms during progression. Based on these findings, the high mTOR (especially mTORC2) activity accompanied by altered metabolic pathways could help cells to survive conventional treatment. These metabolic alterations may serve as therapeutic targets in the future. The detected mTORC1 and C2-related protein expression in our study can elucidate the inefficacy of mTORC1 inhibitor therapy in some cases and could also underline the importance of biomarker-based selection for mTOR inhibitor therapy.

## 4. Materials and Methods

### 4.1. Tissue Microarray Construction and Immunohistochemistry

Tissue microarrays (TMA) (6 × 8 cores, diameter 2 mm) containing double or triple cores per tissue blocks were constructed (3DHistech TMA Master). Representative areas of formalin-fixed paraffin-embedded (FFPE) blocks were selected based on HE stainings as well as Myf-4 and desmin immunoassayed slides by an experienced pathologist. Non-neoplastic liver parenchyma, tonsil, and testis were used as normal tissues and immunohistochemical staining controls in each TMA block. In those cases in which the tumor sample was too small (e.g., core biopsies) or the patients were still undergoing chemotherapeutic treatment, we used whole sections to keep the biopsy materials intact for further diagnostic evaluation.

Immunohistochemistry was performed on 4 µm thick sections. Immunohistochemistry was validated previously in our laboratory. Based on our previous studies, anti-pmTOR (Cell Signaling, Boston, MA, USA, #2976, dilution 1:100, secondary antibody Novolink), anti-pS6 (Cell Signaling, #2211, dilution 1:100, secondary antibody Novolink), and anti-Rictor (Bethyl, A500-002A, dilution 1:1000, secondary antibody Vectastain) antibodies were used to characterize mTOR complex activity in situ [50]. To evaluate mTORC2 activity we did not use pAkt due to technical reasons, but pmTOR and Rictor results were interpreted together to confirm mTORC2 activity. To describe the metabolic features, we applied anti-PFK (Cell Signaling, #8164, dilution 1:100, secondary antibody Novolink), anti-LDHA (Cell Signaling, #3582, dilution 1:400, secondary antibody Novolink), anti-ATPB (Abcam, Cambridge, UK, ab14730, dilution 1:100, secondary antibody Novolink), anti-G6PDH (Abcam, ab133525, dilution 1:100, secondary antibody Novolink), and anti-GLS (Abcam, ab156876, dilution 1:200, secondary antibody Novolink) antibodies.

After deparaffinization and endogenous peroxidase blocking, antigen retrieval was performed for 20–30 min (citrate buffer pH 6). Slides were incubated with the primary and secondary antibodies, followed by DAB (Dako, Carpinteria, CA, USA) chromogen and hematoxylin counterstaining.

The slides were evaluated by two independent investigators. To assess immunoreactions, we used the H-score method. H-score was calculated by multiplying the percentage of positive cells by staining intensity (0, 1+, 2+, 3+). Cases with an H-score higher than 100 were considered positive [51,52].

### 4.2. RICTOR Fluorescence in Situ Hybridization and Droplet Digital PCR for RICTOR Copy Number Variation Analysis

FISH was performed on 4-μm thick sections of FFPE tissue samples, as has been previously described [24]. After deparaffinization and pretreatment, samples were digested at 38 °C for 25 min (Vysis IntelliFISH Pretreatment saline sodium citrate solution and IntelliFISH Protease; Abbott Molecular, Abbott Park, IL, USA). Hybridization was performed at 37 °C for 2 h with *RICTOR* (#*RICTOR*-20-OR; Empire Genomics, Williamsville, NY, USA) and chromosome 5 (Chr5) control (#CHR05-10-GR; Empire Genomics) probes. After post-hybridization washing and air drying, nuclei were counterstained with 4,6-diamino-2-fenilindol (DAPI I, Abbott Molecular). [24] Representative areas were selected based on hematoxylin and eosin slides and were evaluated counting 30 nuclei in at least two different areas of the tumor using a fluorescence microscope. The mean number of the signals per nucleus and the *RICTOR*/Chr5 ratio were determined; *RICTOR* copy number of less than 4 and a *RICTOR*/Chr5 ratio of less than 2 were considered negative, whereas *RICTOR* copy number of 6 or more and a *RICTOR*/Chr5 ratio of 2 or more were considered positive. Cases with a *RICTOR* copy number between 4 and 6 and a *RICTOR*/Chr5 ratio of less than 2 were considered equivocal [24].

Droplet digital PCR was performed for *RICTOR* copy number variation analysis. DNA was extracted and purified from five 10-μm thick sections of the FFPE samples using QIAamp DNA FFPE Tissue Kit (Qiagen, Hilden, Germany) according to the manufacturer’s instructions. For ddPCR, PCR reaction was prepared from 30 ng extracted DNA with *RICTOR* FAM probe (dHsaCNS608884235; Bio-Rad, Hercules, CA, USA) and AP3B1 HEX probe (dHsaCP2500348, Bio-Rad). Droplets were generated using Bio-Rad Automated Droplet Generator (Bio-Rad) device, then emulsified PCR reactions were transferred and run in a 96-well plate on a C1000 Touch thermal cycler (Bio-Rad) (95 °C for 10 min, followed by 40 cycles of 94 °C for 30 s, 60 °C for 1 min, and 98 °C for 10 min). Plates were read on a Bio-Rad QX200 droplet reader and analyzed with using the QuantaSoft software (version 1.2.10; Bio-Rad). A *RICTOR*/AP3B1 ratio of 2 or more was defined as *RICTOR* amplification.

### 4.3. Statistical Analysis

Statistical analysis was performed using IBM SPSS Statistics program version 21 (SPSS Inc., Chicago, IL, USA) using a two-sample t-test, Mann-Whitney U test, Fischer’s exact test, and Spearman rank correlation. We compared the expression of all cases in relation to PAX-FOXO fusion status, time of sampling (primary, during treatment, recurrent), risk stratification, and survival. Risk stratification was performed according to the classification of Intergroup Rhabdomyosarcoma Study Group (IRSG) [26]. *p* < 0.05 was considered as statistically significant.

The study was conducted in accordance with the Declaration of Helsinki, and the protocol was approved by the Ethics and Scientific Committee of Semmelweis University (project identification code: TUKEB 99/2018, approval date: 18.06.2018.).

## 5. Conclusions

Our results confirm the activation of the mTOR pathway in RMSs; moreover, it underlines the significance of the mTORC2 activity. The most important results were the observed high expression of pmTOR in the therapy resistant relapsed cases and the enhanced mTORC2 activity in the studied RMS cases. Our findings highlighted that the expression pattern of the studied mTOR markers can explain the inefficacy of rapalog therapy. Therefore, we suggest performing a detailed investigation of the mTORC1 and C2 profile before administering mTORC1 inhibitor therapy. Furthermore, it was shown that mTOR activation is linked to metabolic adaptation, enhanced glycolysis, pentose-phosphate pathway, and elevated glutamine demand, which can be another cause for treatment failure in this malignancy. Based on our results, targeting the metabolic plasticity of RMS cells could be an alternative therapeutic approach.

## Figures and Tables

**Table 1:** Clinicopathologic characteristics of the studied patients diagnosed with rhabdomyosarcoma (SR, standard-risk; MR, medium-risk; HR, high-risk; No, number).

Histology		Patients
		(*N* = 48)
	Fusion-negative	43
	Embryonal	39
	Botryoid	1
	Spindle cell	3
	Fusion-positive	5
Gender		(No.)
	Female	20
	Male	28
Age		(years)
	Median (range)	5.7 (0–17.3)
Risk group		(No.)
	SR	1
	MR	11
	HR	36
Overall survival		68.8%

**Table 2:** The results of the *RICTOR* amplification analyses. The results of the amplification analysis in the examined 27 cases are presented. We found equivocal result in Case 1 using *RICTOR* FISH analysis, but ddPCR could not confirm *RICTOR* amplification in this case, nor in other studied samples (ND, not determined).

Examined Cases	Rictor H-Score	*RICTOR* FISH (Average *RICTOR*/ Nucleus)	*RICTOR* PCR (*RICTOR*/AP3B1 Ratio)
Case 1	230	equivocal (4.70)	negative (1.47)
Case 37	40	negative (3.90)	negative (0.83)
Other 25 cases	median: 169.00	median: 2.00	ND

## Supplementary Materials

The following are available online at https://www.mdpi.com/2072-6694/12/7/1947/s1. Table S1: The relevant clinicopathological characteristics of the studied patients, Table S2: The mean and median H-score results and the belonging probability values of the primary and recurrent samples according to PAX-FOXO fusion status, risk stratification and survival, Table S3: The immunopositivity and the statistical results of pmTOR, pS6, Rictor, PFKP, LDHA, G6PDH, ATPB and GLS stainings in the studied cases according to PAX-FOXO fusion status, risk stratification and survival.
