# Supplementary material for: Characterization of mTOR Activity and Metabolic Profile in Pediatric Rhabdomyosarcoma
Source: Cancers (Basel). 2020 Jul 17;12(7):1947. doi: 10.3390/cancers12071947 (PMC7409076; doi:10.3390/cancers12071947)
Supplement: Supplementary file 1 [file cancers-12-01947-s001.pdf]

Supplementary Materials

Characterization of mTOR Activity and Metabolic Profile in Pediatric Rhabdomyosarcoma

Luca Felkai, Ildikó Krencz, Dorottya Judit Kiss, Noémi Nagy, Gábor Petővári, Titanilla Dankó, Tamás Micsík, András Khoor, Tamás Tornóczky, Zoltán Sápi, Anna Sebestyén and Monika Csóka

Table S1. The relevant clinicopathological characteristics of the studied patients.

Case No.	Primary	After Preoperative Therapy	Relapse	Fusion Status	Histological Subtype	Risk Group	Gender
1	x			positive	alveolar	HR	male
2	x			negative	spindle cell	HR	male
3	x		x	negative	embryonal	HR	female
4	x			negative	embryonal	HR	female
5	x			negative	embryonal	HR	female
6		x		negative	embryonal	HR	male
7	x			negative	embryonal	HR	female
8	x			negative	embryonal	MR	female
9	x	x		negative	embryonal	HR	female
10	x		x	negative	embryonal	HR	female
11	x		x	negative	embryonal	HR	male
12	x			negative	embryonal	HR	male
13	x		x	negative	embryonal	HR	male
14	x		x	negative	embryonal	MR	female
15	x			negative	embryonal	MR	male
16	x			negative	embryonal	HR	male
17	x			negative	embryonal	MR	male
18	x			negative	embryonal	MR	male
19	x	x		negative	botryoid	HR	female
20	x			negative	embryonal	HR	female
21	x			negative	embryonal	SR	female
22	x			positive	alveolar	HR	female
23	x			negative	embryonal	MR	male
24	x			negative	embryonal	MR	male
25	x			negative	embryonal	HR	male
26	x			negative	embryonal	HR	male
27			x	negative	embryonal	HR	male
28	x		x	positive	alveolar	HR	male
29	x	x	x	negative	embryonal	HR	female
30	x	x		positive	alveolar	HR	female
31	x			negative	embryonal	MR	male
32	x			negative	embryonal	MR	male
33	x		x	negative	embryonal	HR	male
34	x			negative	embryonal	HR	female
35	x			negative	embryonal	HR	male
36	x			negative	spindle cell	HR	male
37	x	x	x	negative	embryonal	HR	female
38	x			negative	embryonal	HR	male
39	x	x		negative	embryonal	HR	female
40	x			negative	embryonal	MR	male
41	x			negative	embryonal	HR	male
42	x	x		positive	alveolar	HR	male
43	x	x		negative	embryonal	HR	male
44	x			negative	spindle cell	HR	female
45	x			negative	embryonal	HR	female
46	x			negative	embryonal	HR	male
47	x			negative	embryonal	MR	male
48			x	negative	embryonal	HR	female

Table S2. The mean and median H-score results and the belonging probability values of the primary and recurrent samples according to PAX-FOXO fusion status, risk stratification and survival. .

		**pmTOR**			**pS6**			**Rictor**			**PFK**			**LDHA**			**G6PDH**			**ATPB**			**GLS**		
	**Total no.**	**mean**	**median**	*p*	**mean**	**median**	*p*	**mean**	**median**	*p*	**mean**	**median**	*p*	**mean**	**median**	*p*	mean	median	*p*	mean	median	*p*	mean	median	*p*
Primary																									
Fusion negative	40	**107**	**120**	0.366	51	38	0.148	**140**	**140**	0.02	43	8	0.214	**110**	**110**	0.985	**139**	**146**	0.597	100	98	0.485	**120**	**137**	0.448
Fusion positive	5	**137**	**143**		16	13		**189**	**193**		79	**103**		**104**	80		**125**	**124**		99	63		**101**	**108**	
Low risk	12	**133**	**153**	0.095	49	58	0.418	**149**	**143**	0.668	30	3	0.185	**132**	**149**	0.126	**142**	**151**	0.626	65	54	0.626	**122**	**129**	0.626
High risk	33	**101**	**115**		46	20		**145**	**150**		53	18		**100**	94		**135**	**133**		83	75		**116**	**135**	
Survived	32	**108**	**121**	0.634	48	32	0.773	**146**	**150**	0.599	44	12	0.723	**114**	**125**	0.475	**141**	**146**	0.288	83	70	0.79	**115**	**129**	0.867
Progressed	13	**115**	**122**		44	30		**138**	**135**		54	10		99	78		**124**	**124**		78	95		**124**	**135**	
Recurrent																									
Fusion negative	9	**170**	**178**	0.099	73	76	0.637	**111**	**105**	0.068	73	58	0.458	**138**	**148**	0.906	86	82	0.813	88	84	0.239	**127**	**143**	0.637
Fusion positive	2	10	10		36	36		**189**	**189**		3	3		**131**	**131**		67	67		21	21		86	86	
Low risk	3	**202**	**202**	0.838	8	8	0.014	**140**	**140**	0.794	58	58	1	**144**	**144**	0.608	67	67	0.609	84	85	0.414	**143**	**143**	0.759
High risk	8	**122**	**125**		80	76		**123**	**121**		59	3		**135**	**148**		86	82		73	43		**113**	**106**	
Survived	3	90	90	0.133	80	80	0.776	**179**	**179**	0.4	60	60	0.63	**115**	**115**	0.497	**108**	**108**	0.776	83	83	0.497	**155**	**155**	0.921
Progressed	8	**150**	**178**		62	60		**114**	**105**		58	13		**142**	**148**		76	82		73	49		**110**	90	

Table S3. The immunopositivity and the statistical results of pmTOR, pS6, Rictor, PFKP, LDHA, G6PDH, ATPB and GLS stainings in the studied cases according to PAX-FOXO fusion status, risk stratification and survival. (H-score < 100 – negative (neg.), H-score > 100 – positive (pos.)).

		**pmTOR**			**pS6**			**Rictor**			**PFKP**			**LDHA**			**G6PDH**			**ATPB**			**GLS**		
	**Total no.**	**Pos. (%)**	**Neg. (%)**	***p***	**Pos. (%)**	**Neg. (%)**	***p***	**Pos. (%)**	**Neg. (%)**	***p***	**Pos. (%)**	**Neg. (%)**	***p***	**Pos. (%)**	**Neg. (%)**	*p*	Pos. (%)	Neg. (%)	*p*	Pos. (%)	Neg. (%)	*p*	Pos. (%)	Neg. (%)	*p*
Primary																									
Fusion negative	40	**55**	45	0.286	18	**82**	0.411	**80**	20	0.357	13	**87**	0.033	**53**	47	0.411	**87**	13	0.643	35	**65**	0.592	**72**	28	0.467
Fusion positive	5	**80**	20		0	**100**		**100**	0		**60**	40		40	**60**		**80**	20		40	**60**		**60**	40	
Low risk	12	**75**	25	0.142	0	**100**	0.094	**92**	8	0.302	8	**92**	0.302	**67**	33	0.26	**92**	8	0.302	25	**75**	0.3	**83**	17	0.223
High risk	33	**52**	48		21	**79**		**79**	21		21	**79**		**48**	52		**79**	21		39	**71**		**66**	34	
Survived	32	**57**	48	0.745	16	**84**	0.68	**81**	19	0.58	16	**84**	0.672	**58**	42	0.347	**84**	16	0.42	34	**66**	0.528	**71**	29	0.589
Progressed	13	**62**	38		15	**85**		**85**	15		23	**77**		**46**	54		**77**	23		38	**62**		**69**	31	
Recurrent																									
Fusion negative	9	**78**	22	0.109	11	**89**	0.818	**50**	**50**	0.333	33	**67**	0.509	**89**	11	0.345	22	**78**	0.491	**56**	44	0.273	**56**	44	0.273
Fusion positive	2	0	**100**		0	**100**		**100**	0		0	**100**		**50**	**50**		**50**	**50**		0	**100**		0	**100**	
Low risk	3	**67**	33	0.721	0	**100**	0.727	**67**	33	0.333	0	**100**	0.339	**100**	0	0.509	0	**100**	0.339	**67**	33	0.424	33	**67**	0.576
High risk	8	**63**	37		13	**87**		**57**	43		38	**62**		**75**	25		38	**62**		38	**62**		**50**	**50**	
Survived	3	33	**67**	0.279	0	**100**	0.727	**100**	0	0.333	33	**67**	0.661	**67**	33	0.491	33	**67**	0.661	**67**	33	0.424	**67**	33	0.424
Progressed	8	**75**	25		13	**88**		**50**	**50**		25	**75**		**88**	13		25	**75**		38	**63**		38	**63**	

© 2020 by the authors. Licensee MDPI, Basel, Switzerland. This article is an open access article distributed under the terms and conditions of the Creative Commons Attribution (CC BY) license (http://creativecommons.org/licenses/by/4.0/).
